# A 2026 horizon scan for biodiversity conservation in South Africa

**DOI:** 10.1007/s13280-026-02365-3

**Published:** 2026-03-13

**Authors:** Colleen L. Seymour, Krystal A. Tolley, Tsungai Zengeya, Dian Spear, Jeran A. Cloete, Anisha Dayaram, Jessica M. da Silva, Graham J. Alexander, Kate Handley, Grant S. Joseph, Lavhelesani D. Simba, Kate Snaddon, Graham P. von Maltitz, Peter J. Carrick

**Affiliations:** 1 South African National Biodiversity Institute, Kirstenbosch Research Centre, Kirstenbosch Gardens, PVT Bag X7, Claremont, 7753 South Africa; 2 Department of Conservation Ecology and Entomology, Stellenbosch University, Stellenbosch, 7602 South Africa; 3 Department of Zoology, University of Johannesburg, Auckland Park, Johannesburg, 2006 South Africa; 4 Centre for Invasion Biology, Department of Zoology and Entomology, University of Pretoria, Pretoria, South Africa; 5 Centre for Sustainability Transitions, Stellenbosch University, Stellenbosch, 7600 South Africa; 6 Restoration and Conservation Biology Research Group, School of Animal, Plant and Environmental Science, University of the Witwatersrand, Johannesburg, South Africa; 7 School of Animal, Plant and Environmental Sciences, University of the Witwatersrand, 1 Jan Smuts Ave, Johannesburg, 2050 South Africa; 8 Biodiversity Law Centre, Kirstenbosch National Botanical Gardens Centre for Biodiversity Conservation, Claremont, 7753 South Africa; 9 FitzPatrick Institute of African Ornithology, Department of Biological Sciences, University of Cape Town, Rondebosch, 7701 South Africa; 10 Department of Biotechnology and Biological Sciences, University of Fort Hare, Alice, 5700 South Africa; 11 Freshwater Research Centre, Imhoff Farm, Kommetjie, 7975 South Africa; 12 Department of Botany and Zoology, Stellenbosch University, Stellenbosch, 7602 South Africa; 13 Institute for Communities and Wildlife in Africa (iCWild), Department of Biological Sciences, University of Cape Town, Rondebosch, 7701 South Africa; 14 Citrus Research International (PTY) Ltd, 2 Baker St, Mbombela, 1201 South Africa

**Keywords:** Biodiversity futures, Emerging issues and step change threats and opportunities, Expert opinion, Social consensus vs. scientific knowledge

## Abstract

Horizon scans identify potential changes, enabling proactive rather than reactive conservation strategies. Here, in a follow up to the 2020 horizon scan, 14 biodiversity professionals from different sectors identify ten emerging issues potentially relevant to biodiversity conservation in South Africa over the next 5–10 years. The issues identified highlight three critical needs: adaptive governance systems, cross-sectoral collaboration capacity, and vigilance around new technologies that may simultaneously offer solutions and create new environmental pressures. We plotted these issues along axes of social agreement and scientific certainty, to ascertain whether issues might be "simple" (amenable to solutions from science alone), "complicated" (socially agreed upon but technically complicated), "complex" (scientifically challenging and condisderable levels of social disagreement) or "chaotic" (high social disagreement and highly scientifically challenging). Only one issue was likely to be addressed with improved science alone, but the remainder were all “complex”, requiring social, economic and political engagement.

## Introduction

Conservation biology is fundamentally concerned with long-term sustainability of biodiversity, yet often operates as a “crisis” discipline, requiring rapid action to prevent species declines before data can be gathered (Soulé 1985). This crisis-driven, reactive aspect has become a defining characteristic of the field (Meine et al. 2006), but new approaches are emerging to address this limitation (Malavasi 2025). This includes a shift from reactive to proactive approaches, allowing better planning for the future (Sutherland and Woodroof 2009). Horizon scanning aims to identify emerging issues that are not yet highly visible, or those about to undergo a marked step change (defined as a considerable and rapid increase in intensity), that could have considerable positive or negative effects on biodiversity conservation. Use of horizon scans and priority setting exercises for biodiversity conservation was pioneered in the late 2000s (e.g., Sutherland et al. 2008, 2009), with annual global horizon scans beginning in 2009 (Sutherland et al. 2010); of the 15 issues identified in 2009, five have become major global issues, and another six increased in importance a decade later (Sutherland et al. 2019).

Horizon scan issues vary in their intensity and impact across different regions, ecosystem types, or taxonomic groups. Countries and regions differ in response options, making regional and national scans valuable (Rudd et al. 2011; Izurieta et al. 2018). South Africa conducted a national horizon scan in 2020 (Seymour et al. 2020). Given that conservation policy, technologies, environmental challenges and socio-economic pressures are constantly changing, we conducted a new horizon scan to identify current priorities for South African biodiversity conservation focused on issues likely to be relevant in the next 5–10 years, and that may inform national and global reporting requirements within similar time periods (e.g. Convention on Biological Diversity, South African National Biodiversity Assessment).

Like many other countries in the Global South, South Africa is megadiverse and hosts three of the world's biodiversity hotspots (Küper et al. 2004). It has a high proportion of threatened species (Raimondo et al. 2023). The country also possesses considerable mineral wealth, which creates employment opportunities but also creates additional threats to the environment (Jewitt et al. 2015). This natural wealth exists within a context of profound socio-economic challenges: extreme inequality, high unemployment, rapid urbanisation, and complex land reform pressures. The country also faces water (Blignaut et al. 2009), energy^1^ and food (van der Berg et al. 2022) insecurity, and has recently seen declining land productivity, exacerbated by drought (Seymour et al. 2025). Here, we (1) identify horizon issues likely to impact South African biodiversity conservation over the next 5–10 years, either new issues or ‘step changes’; (2) categorise these issues in terms of ‘social consensus’ (i.e., agreement) and the need for ‘scientific certainty’, to assess the level of complexity and types of engagement required in response; and (3) contextualise these findings relative to the changing conservation landscape since the time of our last Horizon Scan in 2020.

## Materials and methods

Horizon scanning can be conducted in different ways, ranging from manual expert-based consultation to automated scanning of online content (Wintle et al. 2024). Computational approaches include web-based searches using keywords and search engines (Palomino et al. 2012), text mining to detect signals of potential future changes (Yoon 2012), and machine learning for automated document retrieval and generation of comments (i.e., a short explanation of the contents, opinions, arguments, and/or future expectations) generation (Ishigaki et al. 2022). Each approach has strengths and weaknesses. Although automated systems offer systematic, comprehensive coverage, they need careful keyword selection and can generate results that must be filtered (Palomino et al. 2012). Manual expert-based approaches can access information not yet online, provide contextual understanding through structured deliberation, and identify novel issues for which there are not yet established keywords for automated searching (Wintle et al. 2024). Our approach follows the Delphi-based method, which has been used in annual global conservation horizon scans since 2010 (Sutherland et al. 2010, 2026).

Fourteen biodiversity professionals were selected to represent sectoral and disciplinary diversity. The group included representatives from government biodiversity research institutions (*n* = 7), academia (*n* = 11), non-governmental and nonprofit organisations (*n* = 3), and private consultants (*n* = 3) with several participants holding dual affiliations. Disciplinary expertise included herpetology, entomology, freshwater ecology, systems ecology, conservation planning, restoration ecology, environmental law, biodiversity policy, conservation genetics, biomedical engineering and medicine. This composition was intended to capture diverse perspectives across taxonomic groups, ecosystems, and conservation contexts. Participants were invited based on their active involvement in South African biodiversity conservation, their engagement with policy and management, and their ability to access different professional networks for issue identification. The Delphi method's anonymised approach (where proponents of issues remained unknown during discussion) helped minimise bias arising from institutional affiliation or seniority.

Each participant independently conducted horizon scanning over approximately four months prior to submitting their issues. Participants used multiple approaches to identify potential issues, including consultation with their professional networks and colleagues within their sectors (governmental, non-governmental, academic and private), monitoring relevant media sources (e.g., scientific literature, policy documents, industry reports, environmental news outlets, science journalism, and the media in general), and attention to trends and developments within their own research fields. For four participants who had been involved in the 2020 horizon scan (Seymour et al. 2020), they had also been monitoring emerging trends since that assessment. Participants were asked to focus on issues that were either entirely new or likely to undergo a marked increase in intensity (a ‘step change’). This approach combines systematic environmental scanning with expert knowledge and field experience, allowing capture of both documented trends and emerging concerns not yet widely reported in formal literature.

Participants each submitted 3–4 issues, which were then pooled, generating 51 potential concerns for evaluation. To avoid fatigue bias, four versions with issues in randomised order were created. One version (of the four possible) was randomly assigned to each person in the group. We used a Delphi approach (Dalkey and Helmer 1963), which consisted of three iterative rounds with controlled feedback, maintaining anonymity throughout. For the first round, participants scored every issue out of 1000 points, assigning low scores to familiar, less pressing or unlikely issues and high marks for unknown, emerging issues, or those about to undergo a step change. Every issue was given a unique score to allow ranking. Participants also indicated whether they had heard of an issue before. Scores were converted to ranks and summed across all participants. Anonymity was maintained by not revealing which participant had proposed which issue. This produced a short-list of 24 issues that were circulated to the group, although their relative rankings were not shared, to try to reduce any bias arising from this ranking.

In the second round, the 24 issues were discussed during a seven-hour online meeting in May 2025, following a Delphi-based approach used in previous horizon scans (Sutherland et al. 2010). Each issue was assigned to two participants (who had not proposed that issue) to serve as ‘cynics’ providing in-depth critical assessment. Neither the cynics nor the original issue proponents were identified to the group. The original proponent of each issue did not speak first during discussions to prevent authority bias. The online format (MS Teams) was necessitated by budgetary constraints. All participants kept their cameras on, to minimise difficulties of communicating in online meetings (see Castelli and Sarvary 2021). Ten minutes was allowed for the discussion of each issue, to ensure comparable consideration between issues, timed using a screen ‘countdown’ timer. Participants were allowed to clarify any issues they felt had been misunderstood or to propose new ones that they felt were vital for inclusion, resulting in one additional issue being added (i.e., *n* = 25 issues for the third round).

In the third round, each participant independently scored all 25 issues again out of 1000 points using the same criteria as Round 1. This iteration incorporated the knowledge and perspectives shared during the online discussion while maintaining individual judgement. Scores were again converted to ranks and summed, identifying the 12 highest-scoring issues. The group then agreed to combine four of these into two, owing to substantial overlap, yielding the final ten priority issues.

**Fig. 1:**
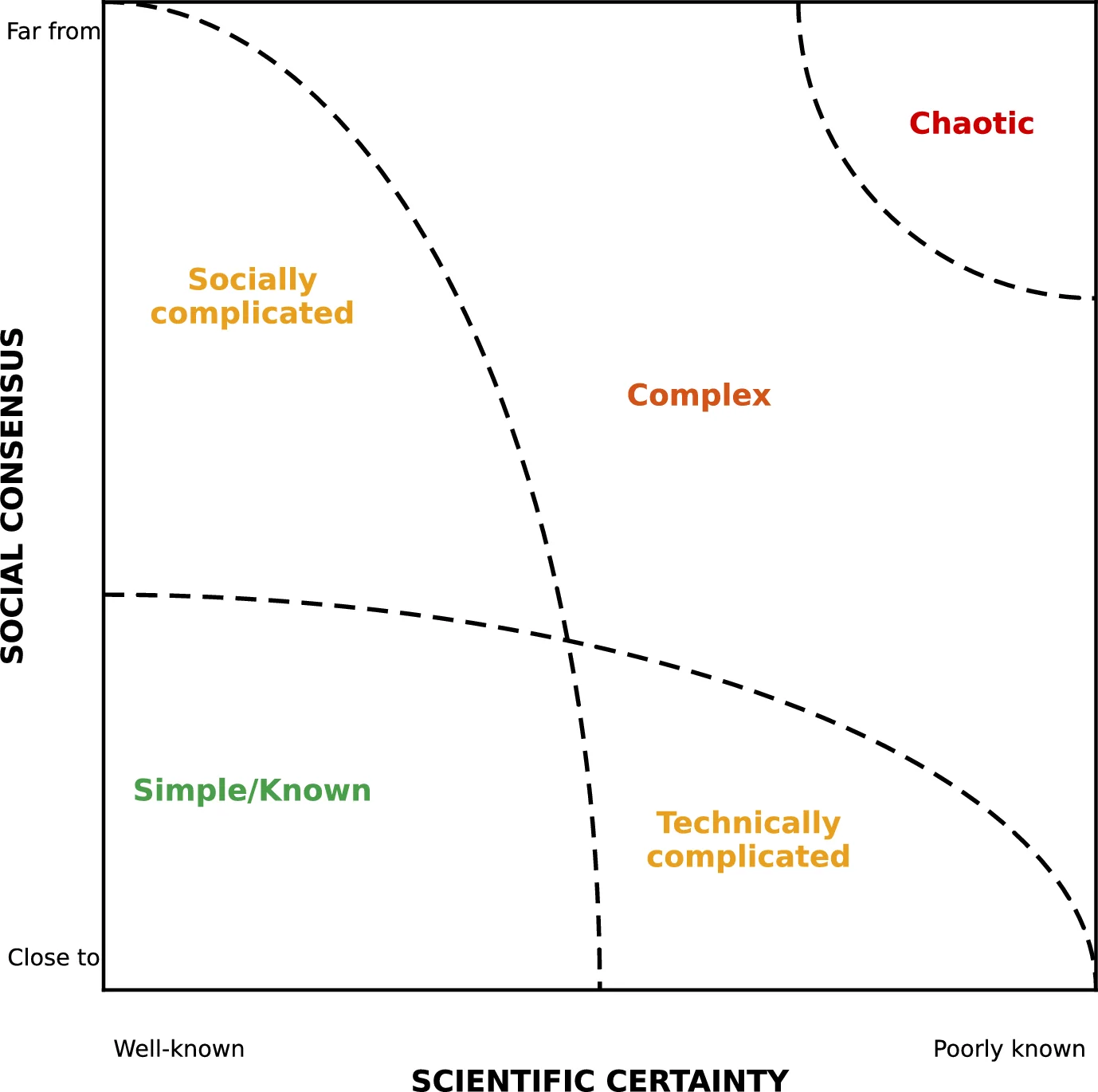
The position of issues along axes of scientific certainty and societal consensus can help researchers, conservation managers and implementers decide on the best way to engage with issues [following Patton (2011)]

We assessed each of the final ten issues along axes of ‘social consensus’ (i.e., agreement) and the need for ‘scientific certainty’, classifying them as ‘simple’, ‘complicated’, ‘complex’, or ‘chaotic’ (Fig. 1). Conservation issues for which social consensus is high are ‘simple’, if they are scientifically well known, or ‘technically complicated’ if they are scientifically uncertain. Many conservation issues exist within complex socio-ecological contexts where outcomes are unpredictable, and stakeholders disagree on priorities and success measures. Science alone cannot resolve ‘complex’ issues; they require integrated scientific, social, economic, and political strategies. Mapping issues along knowledge-consensus axes identifies when broader engagement beyond science becomes essential for effective policy and management. The ‘chaos’ category represents issues where neither consensus nor knowledge exist. Participants rated each issue out of 10 for ‘consensus’ and ‘scientific knowledge’, and we plotted mean scores and standard errors for each dimension.

This multi-round Delphi approach with anonymity and controlled feedback reduces several biases common in expert elicitation exercises (Mukherjee et al. 2015). Anonymization of issue proponents reduces halo effects (where perceptions are influenced by aspects that are irrelevant to the topic) and dominance bias (where group members defer to higher-status individuals). Independent scoring rounds before and after discussion reduce ‘groupthink’, by requiring individuals to form judgments without knowledge of others' opinions. The structured discussion provides controlled feedback that allows participants to revise their assessments based on collective reasoning. The cynic system ensures that each issue receives in-depth independent scrutiny beyond its original advocate.

## Results

Here, we provide a synopsis for each issue, grouped according to themes, and not according to the rank order.

### Future industries and technology

#### Non-aqueous fracking

Energy security is a major concern in South Africa, and a decade ago, shale gas hydraulic fracturing (“fracking”) was promoted as a new energy source, despite environmental risks (Holness et al. 2016). South Africa’s gas-rich shale deposits are located in the water-scarce and biologically relatively unknown Great Karoo, but the fracturing process is water-intensive, which was a primary obstacle to fracking (Scholes et al. 2016). Fracking was ultimately abandoned after it became clear that the gas reserves had been over-estimated (de Kock et al. 2017), commercial benefits would be minimal, and water supply would be problematic. Renewed interest exists in non-aqueous fracturing, which efficiently extracts even small gas reserves, using foam-based proppants (Kalam et al. 2021). This has prompted several government departments to issue new regulations facilitating exploration and extraction of shale gas. This is potentially a marked step change for biodiversity impacts as many of those identified for aqueous fracking are again expected, including habitat fragmentation, infrastructure development and pollutants entering groundwater in a sensitive arid system where ecological recovery would be slow (Todd et al. 2016).

#### Biodiversity and the just energy transition: How green are “green” technologies?

The global push for a just energy transition is driving a step change in demand for critical minerals, such as lithium, cobalt, nickel, copper, manganese, and rare earth elements (UNEP 2024). These materials are essential for renewable energy technologies and are strategic for climate goals (IEA 2021). Additionally, new technologies such as green hydrogen, produced by using renewable energy to split water through electrolysis,^2^ are considered ‘game-changers’ in the transition to a low-carbon economy. South Africa has placed extraction of critical minerals and green hydrogen infrastructure at the forefront of development (Department of Mineral and Petroleum Resources 2025). However, an increase in mining and green hydrogen development poses considerable risks to biodiversity, especially in the arid regions where development is planned, overlapping with sensitive ecosystems that are unlikely to recover easily.^3^ Although aimed at being “green”, aspects of the just energy transition that are damaging to biodiversity should be addressed and minimised.

#### Promise and pitfalls of Artificial Intelligence (AI) applications in field-based conservation

AI applications are undergoing a step change in South African conservation, offering unprecedented monitoring capabilities. For example, AI has been integrated into the citizen science iNaturalist platform to help identify species through machine learning from their photo database. AI can also process camera trap images, with AI software sending alerts to mobile phones when a particular species, or human, is captured in a camera trap (Dertien et al. 2023), which can assist with controlling poaching and mitigating human-wildlife conflict. Passive audio monitoring using AI to identify species of birds, frogs and insects through their calls is being tested in South Africa (Turner et al. 2025). However, these technologies are error-prone and require validation. With declining fieldwork experience among ecologists (Soga and Gaston 2025), accurate validation is not guaranteed. In addition, training datasets can be biased towards common or often-photographed species or create misidentifications between similar-looking species. The requirement for large amounts of training data could systematically exclude rare or cryptic species from AI identification systems, potentially creating blind spots in conservation monitoring. Poorly-considered use of AI tools or incorrect data underlying species’ abundances and distributions could have numerous negative consequences for conservation planning, programmes and funding.

#### User-friendly, but expert-free: Hidden dangers of AI for biodiversity analysis

AI is also undergoing a step change in its use for conservation analysis and planning, and there is likely to be a rapid increase in use for technical applications in biodiversity research and analyses, in particular. Furthermore, AI could soon be the primary tool used by land-use and biodiversity planners, and government regulators. Use of AI for data analysis, and generative AI (GenAI) tools based on large language models (LLMs), have enabled even those with minimal training and limited understanding of the shortcomings of these models to perform complex computational tasks and produce apparently comprehensive reports. This results in outputs from AI being uncritically incorporated into research, planning and Environmental Impact Assessments using AI’s ability to rapidly analyse environmental datasets and synthesise literature. AI model outputs can only be improved with expert assessment of their accuracy, and LLMs are not yet ready for high-stakes scientific applications given that they can often produce “hallucinations”. For example, completely fabricated information was found inserted into 28.6% (ChatGPT 4) to 91.4% (Bard/Gemini) of results when used for systematic medical literature reviews (Chelli et al. 2024). When AI tools extract data from biodiversity publications, systematic biases in studies are amplified and can even be falsely accorded statistical significance. If AI is used unquestioningly, there is a risk of producing superficial outputs, devoid of nuanced local ecological understanding and expert knowledge.

### Infrastructure at landscape scale

#### Negative impacts of utility poles and transmission infrastructure

Biasotto and Kindel (2018) identified 28 negative impacts of utility poles and electricity transmission infrastructure on indigenous species. South Africa faces escalating challenges from three factors: (i) obsolete telephone infrastructure remains without maintenance while cellular communications expand; (ii) immense need for transmission infrastructure in under-resourced areas, and a burgeoning renewable energy rollout^4^; (iii) regulatory streamlining through gazetted transmission corridors that bypass full environmental assessments^5^ (South African Government Notice 113; Government Gazette No. 41445, 16 February 2018). The ecological consequences are already severe. For example, utility poles and pylons provide nesting sites for pied crows (*Corvus albus*) in treeless areas, and the increase in abundance and range of this generalist predator threatens numerous species (Fincham and Lambrechts 2014; Cunningham et al. 2015; Joseph et al. 2017; Tolley et al. 2023). This accumulating infrastructure represents a form of landscape pollution with escalating negative conservation consequences for biodiversity.

#### The green water cycle pushed out of balance by loss of natural vegetation

The water cycle comprises “blue water”, in rivers, wetlands, lakes and aquifers, and “green water”, in soils and plants (Falkenmark and Rockström 2006). In South Africa, there is a will to protect blue water and the ecosystems that supply it. Recognition of the green water component is lacking, however, despite it contributing almost half of South Africa’s precipitation (De Petrillo et al. 2025), making it vital to water security. Notably, approximately 20% of South Africa’s terrestrial evaporation originates from neighbouring countries, particularly Botswana, Zimbabwe, Mozambique and Namibia (De Petrillo et al. 2025). Human activities are having an unprecedented impact on the green water cycle, with potentially dire consequences for water security.^6^ Land conversion is leading to transformation, loss and degradation of vegetation, habitats and soils as green water reservoirs, especially within the strategic water source areas in mesic biomes (where habitat loss has occurred fastest; Skowno et al. 2021) and across neighbouring countries. Vegetation clearing directly threatens South Africa's precipitation, because land conversion disrupts green water flows by altering soil water storage, changing evapotranspiration patterns, and breaking down vegetation-atmosphere feedback loops that sustain regional precipitation patterns. Water resource management should therefore extend to assessment, protection and restoration of green water, within South Africa and across the region (see footnote 6).

### Governance and regulatory challenges

#### Poor regulation and pesticide resistance creating cascading biodiversity impacts

South Africa's pesticide governance failures threaten biodiversity through regulatory inadequacies, overuse, and poor monitoring, resulting in pesticide resistance. For example, after resistance to pyrethroids in *Anopheles funestus* mosquitoes led to a surge in malaria cases between 1996 and 2000, the pesticide DDT (Dichloro-Diphenyl-Trichloroethane) was reintroduced, without establishing resistance monitoring protocols (Wells and Leonard 2006). Recent evidence indicates both *A. arabiensis* and *A. funestus* are developing DDT resistance elsewhere in Africa (Mulamba et al. 2014), and environmental persistence has resulted in DDT bioaccumulation even within protected areas (Wolmarans et al. 2021). Outdated legislation (from 1947), lack of updated pesticide evaluations and outdated pest management ecology (Handford et al. 2015), leaves nearly 200 hazardous pesticides registered for use. Of 86 insecticides in common use in South Africa, 62 are banned in the European Union (Seymour, unpublished data^7^). As Africa's second-largest pesticide user, with climate change intensifying pest pressures and food security concerns, South Africa faces escalating applications across extensive croplands (covering > 15% of the landscape; Skowno et al. 2021). These impacts cascade through ecosystems, eliminating non-target arthropods and their predators (e.g., Bowler et al. 2019; Moreau et al. 2022), disrupting soil ecosystems, and creating feedback loops where declining populations of species that feed on invertebrates necessitates further pesticide intensification.

#### Reinforcing pressures on agriculture: climate change, trade tariffs, and biosecurity

Climate change may force farmers to increase pesticide use (including insecticides, herbicides, and fungicides), as rising temperatures reduce pesticide efficacy, extend pest ranges and speed up breeding cycles (Skendžić et al. 2021), leading to higher pesticide application rates (Matzrafi 2019). Pesticide use in South Africa is not well-regulated, including use of internationally banned or regulated substances. Increased tariffs by the USA on South African imports may produce a shift towards European markets with stricter pesticide regulations (more banned substances and lower residue limits; Hejazi et al. 2022). At the same time, biosecurity regulations designed to reduce the spread of pests currently result in “calendar” spray programs that occur whether or not pests are detected, to guarantee successful pest inspections on exports. Enlarging pest geographic ranges may result in increasingly strict and numerous biosecurity regulations (Waage and Mumford 2008) leading to increased pesticide application and yield and quality losses where alternatives to banned substances such as Mancozeb (a broad spectrum fungicide banned in the EU) are less effective (Cloete et al. 2025). Pesticide use is well established as a driver of biodiversity decline (van Lexmond et al. 2015), so how these tensions play out may have large consequences for biodiversity in South Africa.

#### Locking up of essential biodiversity knowledge behind a wall of regulations

In South Africa, biodiversity research has recently been subjected to a morass of overlapping local, provincial, national and global regulations. Many of these regulations were gazetted decades ago, and intended to regulate commercialisation, guarantee attribution of benefits, curb infectious disease spread, and ensure ethical research. However, regulation alone cannot ensure sustainability of any nation’s natural heritage. Building biodiversity knowledge through scientific research enables high level policy and social engagement, and effective conservation. Yet numerous regulations have been intensified or re-interpreted (Hamer et al. 2021), creating an undiscerning, blanket approach that, although intended to mitigate risk, can, and in some cases, has, halted biodiversity research entirely, despite this research underpinning policy and legislation (Alexander et al. 2021). For example, land-use planning protected area management and inputs to national and international conservation assessments such as the National Biodiversity Assessment, Convention for Biological Diversity, IUCN Red List updates, Biodiversity Management Plans, National Protected Area Expansion Plan, and Key Biodiversity Area delineation all require knowledge on biodiversity. Without this, government cannot respond effectively to biodiversity litigation or emerging conservation crises, owing to a lack of evidence-based support. Given this upswing in restrictive regulation and its risks to research progress, archaic regulations must be urgently reviewed or South Africa risks not only losing knowledge, but research expertise.

#### Biodiversity litigation

Biodiversity litigation is emerging as a powerful strategy for environmental accountability, particularly in response to habitat destruction and failures to uphold conservation obligations (Setzer and Higham 2024). Strategic biodiversity litigation can secure constitutional rights (e.g., Section 24 of the Constitution of South Africa, the right to a healthy environment), and ensures compliance with environmental obligations (Futhazar et al. 2022). Drawing from the success of climate litigation, biodiversity litigation could become a powerful tool for holding governments and corporations accountable for inadequate action. For example, over 230 climate-related cases were filed globally in 2023, shaping climate governance through legal precedent (Setzer and Higham 2024). Biodiversity litigation is following a similar trajectory, with notable biodiversity cases already shaping biodiversity governance. For example, a landmark South African case blocked a proposed coal mine in the Mabola Protected Environment (South Africa), reinforcing the legal status of protected areas (Mining and Environmental Justice Community Network of South Africa and Others v Minister of Environmental Affairs and Others; 2019). Biodiversity litigation has gained impetus through increasing global consensus (e.g., adoption of the Kunming-Montreal Global Biodiversity Framework; CBD 2022), and strong national policy, coming up against stalled implementation of this same policy by government departments. However, litigation can be slow, costly, and inaccessible to under-resourced communities (Bratspies 2021). With growing public awareness, legal capacity, and environmental jurisprudence, South Africa is poised for a step change in biodiversity litigation. As biodiversity loss accelerates and legal tools mature, courts will likely play an increasing role in enforcing environmental protection.

#### Consensus vs. Scientific knowledge

**Fig. 2:**
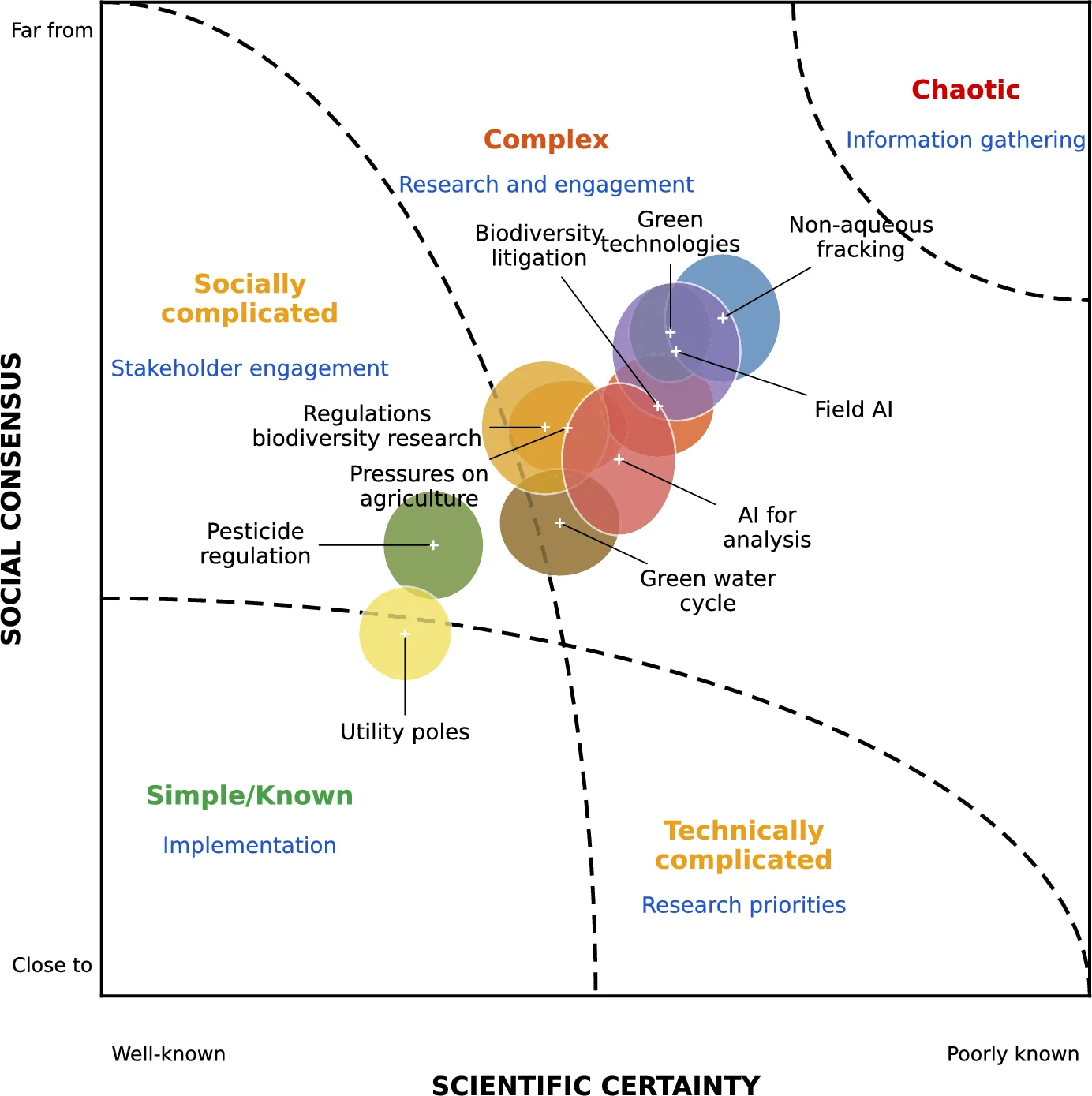
Shortlisted issues mapped onto axes of social consensus (agreement) and scientific certainty. Issues are plotted as ellipses with the centre capturing the mean of votes, and the major and minor axes reflect standard errors for scientific knowledge (x axis), and social consensus (y axis). Scores are between 0 and 10, where 10 represents little to no consensus and little to no scientific certainty. Blue text in brackets beneath the categories indicate the nature of next steps needed. For example, issues that are “socially complicated” require stakeholder engagement, but do not require more research

Most issues fell into the ‘complex’ category, where both science and social consensus are not fully established (Fig. 2). The exception was utility poles, which were considered ‘simple/known’, with well-established science on their impacts and high social consensus about their necessity. Pesticide regulation and its impacts, although falling into the ‘complex’ category, was also considered relatively well-known scientifically, although social consensus remains unresolved across different sectors.

## Discussion

This horizon scan reflects how South African conservation challenges have shifted towards technological disruption, regulatory complexity and infrastructure impacts. Notably, three (fracking, green technologies and utility poles) of the ten issues will impact semi-arid or arid systems in South Africa, which until now have experienced low development pressure. Many issues extend beyond South Africa, given shared characteristics across Africa and other megadiverse developing nations. These challenges highlight three priorities: adaptive governance, cross-sectoral collaboration capacity, and vigilance around new technologies.

### Comparison to conservation challenges identified in 2020

Comparing current issues with those identified five years ago (Seymour et al. 2020) shows some shifts in the conservation landscape. Several themes from 2020 have intensified. For example, concern about "extinction of experience" remains, and is a global challenge. Recent studies find that words relating to nature are used far less frequently in books over time.^8^ Lost experience of nature now manifests alongside AI technologies that could either bridge or widen the gap between people and nature. Infrastructure development pressures highlighted in 2020 continue, including rampant use of game fencing across natural spaces, increased utility poles and infrastructure development.

Our 2020 scan included “zoonotic disease spillover” to humans, just prior to the Covid-19 spillover event. We focused on how a “One Health” approach might encourage conservation, but did not anticipate the global lock-down impacts on ecotourism, with South Africa’s tourism sector losing > 40% of jobs.^9^ This illustrates how horizon scans can identify issues but not fully anticipate outcomes. Unlike 2020, this scan excludes disaster response issues, although these remain important given climate change impacts.

### Technology as both solution and challenge

This horizon scan features technological advancements representing opportunities and risks for biodiversity conservation. For example, AI could offer unprecedented opportunities for biodiversity monitoring, species identification, and conservation planning, but if used uncritically, it poses numerous risks through misidentifications, biased training datasets, and crucially, oversimplified characterisation of ecological systems and threats to them. Similarly, technologies essential for climate mitigation, such as green energy infrastructure, may pressure biodiversity through increased demand for critical resources. New technologies for non-aqueous fracking demonstrate how innovation can revive previously constrained extractive practices, and that we should remain vigilant about new technologies opening new threats to biodiversity.

### Landscape scale infrastructure

Several issues reflect new environmental pressures, or new awareness of long-existing pressures. Although humans have disrupted green water cycles for centuries, recognising this disruption represents a fundamental shift in understanding ecosystem services. Although known for a couple of decades, less than half of our group (47%) were familiar with it. Increased recognition of this issue is urgently needed to ensure that risks to the green water cycle are incorporated into land use and conservation planning and management, locally and regionally.

Major conservation impacts are envisaged with the rapid change in energy provision compounded by continued presence of older infrastructure. The increase in the number of sites of energy generation, including green hydrogen, and more extensive transmission infrastructure, have direct, compounding and far-reaching impacts through trophic cascades. A 2020 theme “Foreign global development goals could threaten local biodiversity” resurfaces. Green energy and fracking developments, often by foreign companies,^10^ and promising jobs, pose biodiversity risks despite economic appeal. Environmental Impact Assessments (EIAs) are necessary to assess the feasibility of these activities, but are often rushed and/or superficial, or only look at mitigating impacts, particularly when in Special Economic Zones (which have lower EIA thresholds). The conservation sector must watch these developments, particularly poorly considered AI use in EIAs.

### Regulatory barriers and monitoring gaps

The emerging pressures identified above are exacerbated by regulatory challenges. Bureaucratic barriers to biodiversity research reflect a concerning trend where well-intentioned regulations may hamper scientific knowledge needed for effective conservation, limiting research that informs protection strategies, and discouraging field-based research, eroding ecological field experience (Soga and Gaston 2025). At the same time, global trends towards increased understanding and regulation of large-scale biological impacts, often driven by legitimate concerns about biopiracy, disease transmission, and ethical research practices, provide a framework for biodiversity litigation. The challenge lies in developing regulatory frameworks that protect biodiversity whilst enabling foundational research for conservation.

### Complexity and the need for social engagement

As in the previous South African horizon scan (Seymour et al. 2020), most identified issues fall within the ‘complex’ category of our knowledge-consensus framework (Fig. 2), where scientific uncertainty intersects with social disagreement. This complexity is evident across different types of issues. Utility poles fell into the ‘simple/known’ category because their impacts are well-known (Biasotto and Kindell 2018), and that most sectors agree they are needed, with a focus on mitigating their presence. Pesticide regulation and its impacts overlapped with the ‘socially complicated’ zone because although negative effects are well known, different sectors (agriculture, conservation and health sectors) disagree on their required levels or use.

That said, most South African conservation issues require collaborative approaches spanning scientific, social, economic, and political domains. Complex issues highlight how biodiversity challenges are embedded within broader socio-economic contexts in developing nations. Issues such as biodiversity litigation, agricultural pressures from climate change and trade policies, regulatory impediments to research, and monitoring of pesticide use, demonstrate how conservation science must engage with legal, economic, governance systems and indigenous knowledge (Joseph et al. 2024; Roy et al. 2024).

### Implications for conservation

Three priorities manifest clearly across our findings. Firstly, the governance imperative is evident in issues around bureaucracy, weak implementation and monitoring, which highlight the need for systems that rapidly respond to technological and environmental change while maintaining long-term conservation objectives. Several issues require immediate action. Pesticide governance demands urgent legislative review. South Africa's 1947 Act and absence of resistance monitoring protocols cannot protect biodiversity under current agricultural intensification. For example, research institutions could partner with the Department of Agriculture to establish or strengthen baseline resistance monitoring for key pest species, and conservation NGOs could work with farming communities to educate and pilot integrated pest management approaches that reduce reliance on toxic substances so as to minimise harm to both the environment and human health. Consideration of disruptions to the green water-cycle needs to be integrated into water resource and land-use planning frameworks, both nationally and regionally. Practically, this could involve hydrologists and soil scientists collaborating with land-use planners to set vegetation-retention thresholds for development authorisations in strategic water source areas, or terrestrial ecologists working with regional catchment management agencies to monitor evapotranspiration rates across southern Africa. These kinds of collaborations need the support and improved coordination between the various national Departments (i.e., Forestry, Fisheries and Environment [DFFE], Water and Sanitation [DWS], and Agriculture [NDA]). Secondly, many issues were complex, implying that conservation organisations may need capacity to engage with fields ranging from artificial intelligence to international trade law. This can likely only be achieved through collaboration, continued public engagement and education. Finally, the conservation sector needs training in recognising AI generated reports, and must remain watchful about new and emerging technologies, and indiscriminate use of AI, which is particularly important for decision-making. The use of LLMs in completing EIAs means that regulations need urgent revision. These should require disclosure of LLM use, and submission of the AI prompt history for audit. Regulations should also specify how they can be used to prevent the introduction fabrications (e.g. limiting LLM use to rewriting original content for improved clarity). Linguistic experts could develop practical guidelines for EIA reviewers on how to identify common chatbot language, formatting and phrase patterns, so that undisclosed use of AI can be detected. Applications of AI, such as generating species lists and impact predictions, will need validation protocols to be developed before they can be used in EIAs.

### Horizon scanning for strategic foresight

Horizon scanning provides early warning of emerging issues, but represents only the first step in strategic conservation planning (Wintle et al. 2024). Translating scanning outputs into effective responses requires understanding the nature of each issue and the type of intervention needed. Our consensus-certainty framework (Fig. 2) categorises issues to guide this translation. ‘Simple’ issues, where both social agreement and scientific understanding are high, primarily need effective implementation of known solutions. ‘Technically Complicated’ issues, where consensus exists but scientific understanding is limited, point to research priorities, and ‘Socially Complicated’ issues are where scientific understanding is well established, but social consensus is low. ‘Complex’ issues, characterised by low consensus and only moderate knowledge, require stakeholder engagement and negotiation before technical solutions can be implemented. ‘Chaotic’ issues, where neither consensus nor scientific certainty exist, need rapid information gathering, monitoring, and stakeholder dialogue to establish next steps.

Although ranking was essential for identifying our top 10 priority issues, we present them grouped by themes rather than in rank order. All 10 issues reached our priority threshold, and presenting them by rank could misleadingly suggest lower-ranked issues are less urgent or disproportionately focus attention on the highest-ranked issue at the expense of others. The final ranking also carried considerable uncertainty given scoring variability across participants. Thematic grouping provides a more useful organising framework for understanding how issues relate to each other and to policy domains, and our consensus-certainty framework indicates what type of response each issue needs.

This structured approach to horizon scanning, in which issue identification is combined with strategic categorization, can help conservation organisations to prepare for emerging challenges. Regular horizon scanning, coupled with monitoring of how identified issues develop over time, can build organisational capacity for strategic foresight. Such capacity becomes increasingly critical as conservation challenges grow more complex and interconnected, requiring proactive rather than reactive responses.

### Looking forward

This horizon scan should not be a one-off exercise, but the first step in strategic foresight. We propose regular reviews (approximately every 5 years) to track how identified issues progress, with findings informing ongoing conservation planning processes. Group composition likely influenced which issues were identified, and our assessment of social consensus versus scientific knowledge. Future scans could benefit from including representatives from the agricultural sector, indigenous and local knowledge holders, and industry stakeholders to broaden perspectives on emerging conservation challenges.

Each scan captures challenges at the specific moment of expert consultation. Horizon scans aim to expand preparedness for possible futures, rather than to make perfect predictions. Our 2020 scan identified zoonotic disease risks prior to the Covid-19 pandemic, although we failed to consider how a zoonotic pandemic might influence conservation. Nevertheless, this does demonstrate the value of anticipating challenges and the need for ongoing monitoring. This scan reveals how multiple pressures will exacerbate conservation challenges. Strategic responses to the issues identified will need coordination between research, regulation, and implementation. The findings should enable concrete change. For example, addressing pesticide governance failures, alerting authorities to AI use in EIAs, and raising broader awareness of these emerging issues within the conservation sector. Early identification enables development of needed partnerships and frameworks spanning technological, regulatory, and social systems.

## Data Availability

There are no data associated with this paper.

## Footnotes

1 https://earth.org/energy-crisis-south-africa/.

2 https://tinyurl.com/37carcke.

3 https://tinyurl.com/3rmcvp64.

4 https://tinyurl.com/2e73jfak.

5 https://tinyurl.com/3pt37k3z.

6 https://watercommission.org.

7 https://tinyurl.com/324cbzpf/.

8 https://tinyurl.com/4c2kkf9z.

9 https://www.statssa.gov.za/?p=16182.

10 https://tinyurl.com/4nmsfkm3.
